# Shedding Light on the Role of ERAP1 in Axial Spondyloarthritis

**DOI:** 10.7759/cureus.48806

**Published:** 2023-11-14

**Authors:** Mohamed A Saad, Amal B Abdul-Sattar, Ibrahim T Abdelal, Ahmed Baraka

**Affiliations:** 1 Rheumatology and Rehabilitation, Physical Medicine and Rehabilitation (PMR) Hospital, Kuwait, KWT; 2 Rheumatology and Rehabilitation, Faculty of Medicine, Zagazig University, Zagazig, EGY; 3 Clinical Pathology, Faculty of Medicine, Zagazig University, Zagazig, EGY

**Keywords:** disease activity, single nucleotide polymorphism, hla-b27, genetics, erap1 gene, ankylosing spondylitis, axial spondyloarthritis

## Abstract

Spondyloarthritis (SpA) is a multifactorial chronic inflammatory disease affecting the axial skeleton (axSpA) and/or peripheral joints (p-SpA) and entheses. The disease's pathogenesis depends on genetic, immunological, mechanical, and environmental factors. Endoplasmic reticulum aminopeptidase 1 (ERAP1) is a multifunctional enzyme that shapes the peptide repertoire presented by major histocompatibility complex (MHC) class I molecules. Genome-wide association studies (GWAS) have identified different single nucleotide polymorphisms (SNPs) in ERAP1 that are associated with several autoimmune diseases, including axSpA. Therefore, a deeper understanding of the ERAP1 role in axSpA could make it a potential therapeutic target for this disease and offer greater insight into its impact on the immune system. Here, we review the biological functions and structure of ERAP1, discuss ERAP1 polymorphisms and their association with axSpA, highlight the interaction between ERAP1 and human leukocyte antigen (HLA)-B27, and review the association between ERAP1 SNPs and axSpA clinical parameters.

## Introduction and background

Axial spondyloarthritis (axSpA) is a chronic autoimmune musculoskeletal disorder primarily affecting the skeletal system. Peripheral manifestations (arthritis, enthesitis, and dactylitis) and extraskeletal manifestations are frequent, with the latter referring to acute anterior uveitis, inflammatory bowel disease, and psoriasis. It ranges from non-radiographic axSpA to radiographic axSpA and is also recognized as ankylosing spondylitis (AS) [1].

Axial spondyloarthritis is an unresolved rheumatic disease that results in bony ankylosis, pain, and functional limitations, primarily affecting the lumbar spine and the sacroiliac and peripheral joints [2]. The quality of life of patients with axSpA gradually deteriorates with disease progression. As a result, patients lose their ability to work and care for themselves, burdening society and the patient's families. It is one of the most challenging diseases to treat, with a significant risk of impairment and a high cost of care [3].

Chronic inflammation causes pain and spinal ankylosis in axSpA. However, the mechanisms underlying this chronic inflammation remain unclear. Despite years of research on the complexities of axSpA, little progress has been made in identifying the signaling events that lead to disease development [4].

Recent advances in our understanding of axSpA pathogenesis have resulted in a better understanding of risk factors, disease causation, and the development of targeted treatments [5]. Genetic studies have significantly improved the understanding of axSpA [6]. The pathogenic mechanisms of axSpA include a complex interaction between the genetic background, environmental triggers, and mechanical stress, resulting in the overall initiation of inflammation and autoimmune reactions [7].

Genetic susceptibility to axSpA is highly complex, as demonstrated by several genome-wide association studies (GWAS) [8]. Hundreds of genes, primarily immune-related, have been identified to be associated with the axSpA spectrum [9]. After the identification of human leukocyte antigen (HLA)-B27 in 1973 as a significant genetic risk factor, its contribution to axSpA evolution became known for the first time [10-12]. This link was so strong that HLA-B27 was thought to be the sole genetic factor that predisposed individuals to axSpA [13]. This particular allele is shared by 85% to 90% of patients with axSpA, even though only 5% of people carrying HLA-B27 in their genetic background will develop axSpA [14]. Over time, many efforts have been made to understand the mechanism by which major histocompatibility complex (MHC) class I molecules interfere with axSpA pathogenesis. Therefore, three different theories have been proposed to explain the role of HLA-B27: (1) arthritogenic peptide, (2) HLA misfolding and accumulation, and (3) HLA-B27 homodimers on the cell surface [15].

The involvement of other non-HLA MHC genes, such as MICA, TNF, transporter associated with antigen processing (TAP)1, TAP2, and LMP2, has also been suggested but has not been confirmed because of linkage disequilibrium with HLA-B27 [16]. The development of GWAS has recently resulted in the identification of additional non-MHC susceptibility loci for axSpA, two of which, namely endoplasmic reticulum aminopeptidase 1 (ERAP1) and interleukin 23 receptor (IL23R), are particularly interesting because they shed light on the important biological pathways involved in SpA pathogenesis [17].

Further, axSpA-associated genetic pathways include other cytokines, kinases, and transcriptor factors with a critical role in inflammation, such as STAT3, TYK2, JAK2, TNF, IL-1, and CARD9 [18]. Additionally, genetic studies have linked axSpA to CD8+-associated factors, such as T-box transcription factor 21 (TBX21), eomesodermin (EOMES), and runt-related transcription factor 3 (RUNX3) [19].

**Figure 1:**
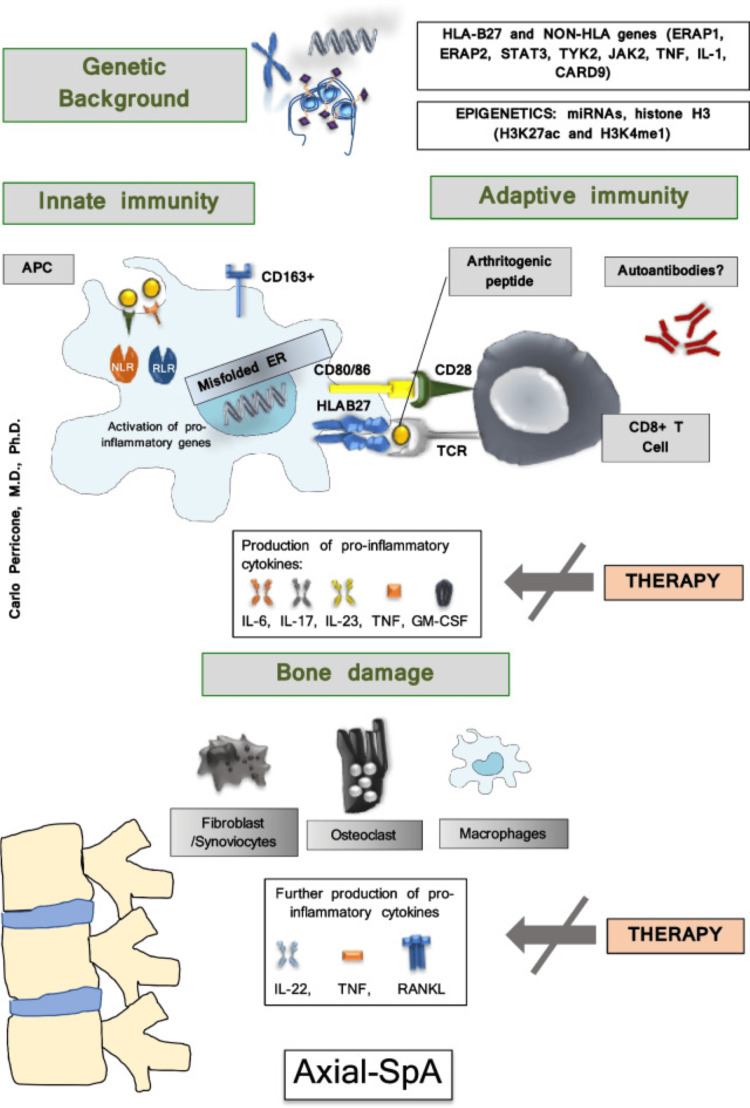
Multi-step pathogenesis of axSpA axSpA: Axial spondyloarthritis, HLA: Human leukocyte antigen, TNF: Tumor necrosis factor receptor Figure adapted from Fatica et al. [17]. This article is licensed under a Creative Commons Attribution 4.0 International License, which permits unrestricted use, distribution, and reproduction in any medium, provided the original author and source are credited.

Genetic variants, as well as epigenetic mechanisms such as DNA methylation, histone modification, and noncoding RNAs, are particularly relevant in explaining SpA pathogenesis. Alterations of histone H3 (H3K27ac and H3K4me1) seem to be correlated with RUNX3 expression and the reduction of CD8+ T cells in the presence of the rs4648889 SNP variant in patients with SpA [20]. Among epigenetic mechanisms, microRNAs (miRNAs) are the most intriguing [17]. The pathogenesis of axSpA is summarized in Figure 1 [17].

## Review

Biological functions of ERAP

The human cellular immune system detects damaged and infected cells based on the exposure of peptides produced by proteolytic processing of intracellular and endocytosed proteins, including aberrant and unnecessary proteins, on their surface. The immune response is triggered by the binding of these potentially immunogenic peptides to MHC class I molecules expressed on all nucleated cells and platelets and presented to CD8+ T lymphocytes [21].

Both ERAP1, ERAP2, and insulin-regulated aminopeptidase (IRAP), an enzyme expressed in endosomes with a peptide trimming role analogous to that of ERAP1/2, are components of the M1 zinc metalloproteases, specifically the oxytocinase subfamily. Endoplasmic reticulum aminopeptidase 1 shares 49% and 43% sequence homology with ERAP2 and IRAP, respectively, mainly within conserved active site domains [22]. Although ERAP1 is expressed in both humans and rodents, ERAP2 is absent in rodents and is not expressed as a full-length protein in approximately 25% of the human population, despite being present in the human genome [23,24], suggesting that its role at any rate is dispensable [25].

The peptide repertoire of cells is sustained through the antigen processing and presentation pathways. This mechanism permits the production of various ideal peptides for MHC class I molecules [26]. The presentation process is the result of a sequence of steps. These peptides are shaped through the antigen processing machinery (APM) [21]. The earlier stages of antigen presentation begin in the cytosol, where the proteasome or immunoproteasome undertakes the first processing event under inflammatory conditions. Abnormal proteins are degraded by the ubiquitin-proteasome system, which induces the generation of smaller peptide fragments. The proteasome/immunoproteasome cleavage pattern often results in a hydrophobic C-terminal residue that is optimal for loading into the F-pocket of most MHC class I peptide-binding grooves [22]. The resulting peptides are transported to the ER via a TAP protein complex [27].

The selection of antigenic peptides that bind to MHC class I molecules is a critical step in MHC class I maturation in the ER [28]. MHC class I attaches its peptide load to the endoplasmic reticulum (ER) via a peptide-loading complex. While MHC class I tends to attach peptides between eight and 11 amino acids long (the majority of which are 9 mers), multiple peptides that enter the ER can be substantially longer. Both ERAP1 and ERAP2 are two ER-resident aminopeptidases that catalyze precursor peptides and define the peptide pool available for binding to MHC class I [29].

**Figure 2:**
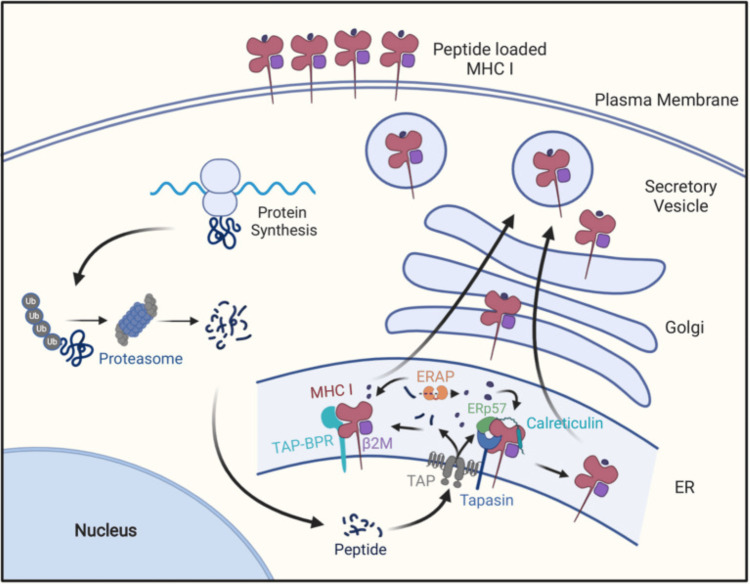
MHC class I antigen presentation pathway Cellular proteins are hydrolyzed by the ubiquitin-proteasome pathway into oligopeptides, which are subsequently transported into the ER through the TAP transporter. In the ER, these peptides may be further trimmed by ERAP1, and then peptides of the right length and sequence bind to MHC class I molecules with the help of tapasin in a peptide-loading complex containing tapasin, TAP, calreticulim, and ERP57, or with the help of TAPBPR. After MHC class I molecules bind peptides, they are transported to the cell surface for display by CD8+ T cells [30]. ER: Endoplasmic reticulum, MHC: Major histocompatibility complex, TAP: Transporter associated with antigen processing Figure adapted from Dhatchinamoorthy et al. [30]. This article is licensed under a Creative Commons Attribution 4.0 International License, which permits unrestricted use, distribution, and reproduction in any medium, provided the original author and source are credited.

Finally, MHC class I antigen processing and presentation pathways (Figure 2) are completed with the help of molecules and chaperones, namely, the peptide loading complex (PLC), TAP, tapasin, ERp57, and calreticulin, which result in the formation of stable peptide-loaded MHC class I molecules that can egress to the cell surface for the expression of CD8+ T cells and NK [29].

**Figure 3:**
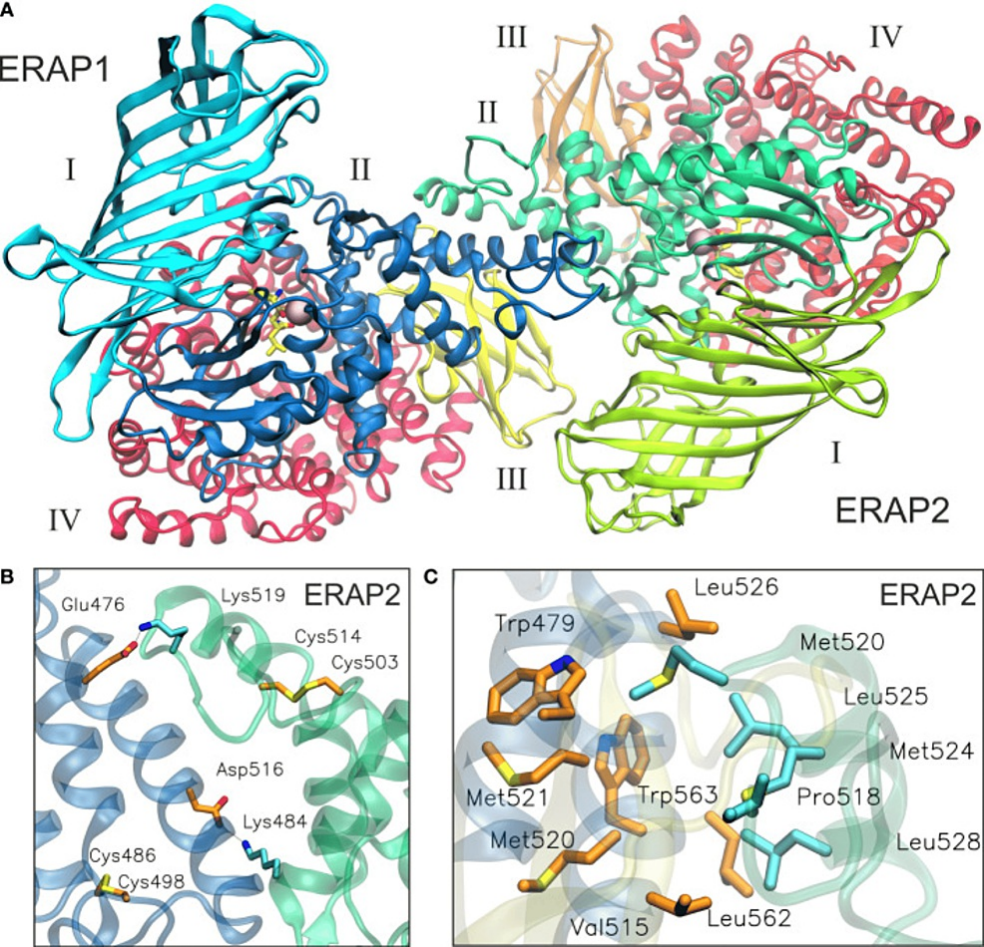
Illustration of the heterodimeric ERAP2/ERAP1 model A: The proposed heterodimeric ERAP2/ERAP1 model B2 from a representative snapshot taken from a 100-ns MD simulation. The snapshot is the centroid of the highest-populated cluster of conformations, representing 64% of the trajectory within a 2 Å RSMD of all Cα atoms. B: Close-up view showing the two key salt-bridge interactions between helix 8 of ERAP1 (orange C atoms) and ERAP2 (cyan C atoms). Disulfide bridges that stabilize exon 10 loops are shown as sticks. C: Close-up view of the dimeric interface illustrating the hydrophobic/aromatic interactions between ERAP1 (orange C atoms) and ERAP2 (cyan C atoms) [35]. ERAP: Endoplasmic reticulum aminopeptidase Figure adapted from Papakyriakou et al. [35]. This article is licensed under a Creative Commons Attribution 4.0 International License, which permits unrestricted use, distribution, and reproduction in any medium, provided the original author and source are credited.

Therefore, by modifying ERAP1 and ERAP2 activity and/or expression, ERAP SNPs have a pronounced influence on the availability and repertoire of antigenic peptides for presentation by HLA class I molecules [31], thereby influencing disease vulnerability [32]. Although these two enzymes may function individually, they may also form a heterodimer to enhance trimming efficiency. The protein encoded by ERAP1 acts as a monomer or heterodimer with ERAP2 [33]. Endoplasmic reticulum aminopeptidase 1 is believed to be the central enzyme in the ER that is involved in peptide trimming, whereas ERAP2 plays a minor role [34]. Figure 3 illustrates the heterodimeric ERAP2/ERAP1 model.

Endoplasmic reticulum aminopeptidase 1 not only plays a canonical function in the adaptive immune system through its function in the ER as an aminopeptidase processing peptide destined for MHC class I presentation to CD8+ T cells, but is also needed to repress several pro-inflammatory and innate immune responses [36]. It also plays a role in the proteolytic cleavage of cytokine receptors, such as tumor necrosis factor receptor 1 (TNFR1), IL6R2, and IL1R2, which are expressed on the cell surface via receptor cleavage. The shedding of cell surface receptors by ERAP1 can control the cellular immune response by modulating receptor availability on the cell surface, which causes a reduction in proinflammatory signaling [37].

Human ERAP1 variants enhance IL-1β production by human immune cells via a mechanism that implicates K+ efflux, a well-known signal for the nucleotide-binding domain, leucine rich-containing family, and pyrin domain-containing-3 (NLRP3) inflammasome activation. However, the mechanisms underlying the ERAP1-dependent immune responses remain unknown. The synthesis of inflammatory cytokines and chemokines by the innate immune system is mainly mediated by the activation of different germline-encoded pattern recognition receptors (PRRs), such as TLRs, RIG-I-like receptors (RLR), and NOD-like receptors (NLRs). The activation of these PRRs causes the coordinated activation of intracellular signaling pathways that regulate the transcription of chemokine genes, inflammatory cytokines, and other innate immune defense reactions [36].

**Figure 4:**
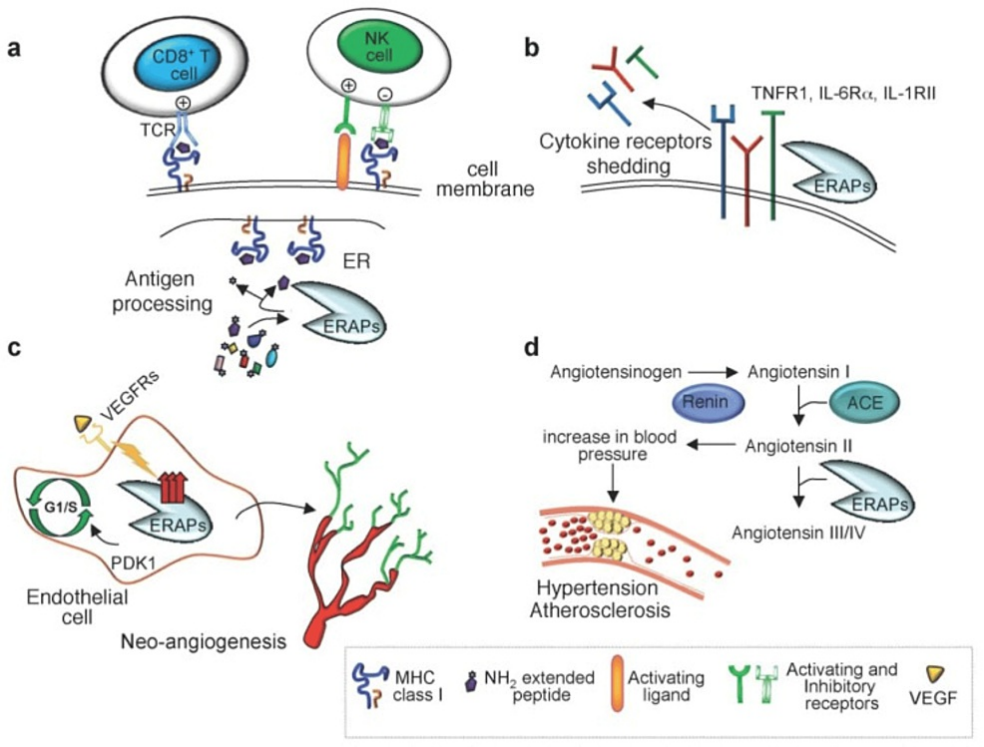
Illustration of various functions of ERAP1 and ERAP2 Endoplasmic reticulum aminopeptidases play diverse roles in various biological processes, including (a) the final step in peptide trimming in the ER for presentation on MHC class I molecules; (b) the shedding of several cytokine receptors; (c) postnatal angiogenesis; and (d) the regulation of blood pressure [40]. ERAP: Endoplasmic reticulum aminopeptidase, ER: Endoplasmic reticulum, TNFR1: Tumor necrosis factor receptor 1, ACE: Angiotensin-converting enzyme, PDK1: Pyruvate dehydrogenase kinase 1, VEGFRs: Vascular endothelial growth factor receptors, MHC: Major histocompatibility complex Figure adapted from Wu et al. [40]. This article is licensed under a Creative Commons Attribution 4.0 International License, which permits unrestricted use, distribution, and reproduction in any medium, provided the original author and source are credited.

In addition to being involved in the immune system, ERAPs contribute to cell migration and angiogenesis, which are essential processes in both pregnancy and cancer [38]. The lack, or downregulation of ERAP1 expression upsets the antigen-presenting properties and immunological function of MHC class I molecules in the host defense against infection. Evidence for the role of ERAP1 in regulating blood pressure comes from in vitro studies showing that this enzyme inactivates angiotensin II through its conversion to inactive angiotensin IV and converts kallidin to bradykinin [39]. Figure 4 shows the functions of ERAP1 and ERAP2.

Structure of ERAP1

Endoplasmic reticulum aminopeptidases are located in the short arm of chromosome 5q15 in a 167Kb region [41]. They share two fundamental sequence motifs vital for enzymatic activity: HEXXH(X) 18E zinc binding and GAMEN substrate recognition sequences. Alternative splicing of ERAP1 produces two N-glycosylated isoforms: ERAP1a (948 amino acids and 20 exons) and ERAP1b (941 amino acids and 19 exons). Endoplasmic reticulum aminopeptidase 1b is more frequent than ERAP1a [42], and they have similar amino acid arrangements, except for some amino acids at the C-terminal and several 3’untranslated regions (3’UTR) sequences [43]. Both ERAP1 and ERAP2 have a similar configuration, comprising four structural domains arranged in a concave orientation around the active site [35].

The crystallographic configuration of ERAP1 revealed four domains: domain 1 (46-254 residues), domain 2 (255-529 residues), domain 3 (530-614 residues), and domain 4 (615-940 residues), which form the final structure of ERAP1 [42]. The structural domains are positioned in a concave orientation around the active site. Endoplasmic reticulum aminopeptidase 1 has been crystallized in two distinct conformations, in which the relative arrangement of the four domains is modified to either reveal or shelter a large internal cavity from the external solvent [44].

One of the crystal structures matched the closed conformation observed in other members of the M1 aminopeptidase family. The other two crystal structures captured an open conformation in which domain IV underwent rigid-body translocation away from domain II, revealing the internal cavity to the bulk solvent. Along with the reorientation of domain IV, the active site was rearranged with tyrosine 438, rotating away in a position unsuitable for catalysis. This mechanism proposes a relationship between conformational state and catalytic activity [45]. Domain III acts as a hinge, allowing open-close-open transitions [46]. This internal cavity can adapt to large peptide substrates, and the conformational change between these two states is crucial for catalytic activity [45]. Endoplasmic reticulum aminopeptidase 2 has only been crystallized in a “closed” conformation in which the internal cavity is not accessible to the external solvent, thus making a conformational change similar to that observed in ERAP1 obligatory for product-substrate exchange [47].

The rs30187, which encodes the K528R variant, and the rs27044, which encodes the Q730E variant, are SNP variants [48] located in the ERAP1 regulatory domain (ERAP1_R), which is separated from their catalytic N-terminal domain. Endoplasmic reticulum aminopeptidase 1 favors peptide substrates with C-terminal hydrophobic residues, which have been demonstrated to anchor to a hydrophobic pocket on the binding surface of ERAP1_R [49].

Endoplasmic reticulum aminopeptidase 1 is highly polymorphic, with multiple common isoforms occurring in the general population. Functional examination revealed that ERAP1 missense SNPs affect the trimming efficiency of specific substrates [32]. Several ERAP1 SNPs are also strong expression quantitative trait loci (eQTLs) [50]. Endoplasmic reticulum aminopeptidase 1 haplotype combinations include more evident vulnerability consequences [51].

The rs2287987 polymorphism is located at the active site; rs30187 and rs10050860 are located at domain junctions; and rs27044 and rs17482078 are located on the inner surface of the peptide-binding cavity of ERAP1 [52]. These polymorphisms influence the substrate specificity and catalytic activity of ERAP1 in a substrate-dependent manner [53].

Endoplasmic reticulum aminopeptidase 2 has only one missense SNP, rs2549782, with apparent discrepancies in the trimming efficiency between the two SNP alleles. This SNP is in ideal linkage disequilibrium with rs2248374, a splice-site SNP that influences ERAP2 splicing and results in a transcript isoform with an extended exon-10 region having a premature stop codon. The powerful eQTL effect observed for this SNP can be explained by the degradation of the ERAP2 transcript through the nonsense-mediated decay pathway [32].

**Figure 5:**
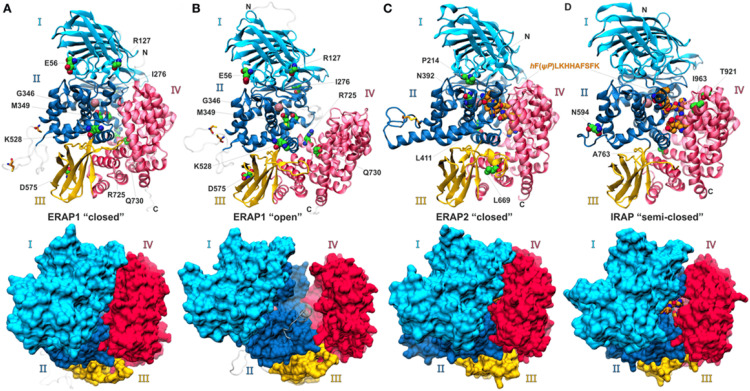
Ribbon and surface representations of ERAP1, ERAP2, and IRAP A and B: ERAP1 complexes with the aminopeptidase inhibitor, bestatin, in the "closed" and "open" states; C: ERAP2, and D: IRAP complexes with a phosphinic pseudopeptidic inhibitor that is shown with orange-colored spheres. The four domains are labeled and color-coded as cyan, blue, yellow, and red for domains I, II, III, and IV, respectively. Catalytic zinc is shown as a pink sphere, and polymorphic site residues are indicated by green spheres. The modeled regions in ERAP1 that were not determined in the X-ray structures are shown in gray [56]. ERAP: Endoplasmic reticulum aminopeptidase, IRAP: Insulin-regulated aminopeptidase Figure adapted from Papakyriakou and Stratikos [56]. This article is licensed under a Creative Commons Attribution 4.0 International License, which permits unrestricted use, distribution, and reproduction in any medium, provided the original author and source are credited.

While ERAP1 and ERAP2 exhibit similar overall structures and mechanisms, they show significant differences, suggesting distinct roles in antigen processing. They have specific preferences for N-terminal amino acids (ERAP1 favors hydrophobic amino acids, whereas ERAP2 favors positively charged amino acids) and differ in their preference for substrate length (ERAP1 prefers peptides longer than 9 amino acids, while ERAP2 can efficiently trim shorter peptides) [44]. Additionally, these enzymes have distinct effects on the cellular immunopeptidome, possibly due to differences in their internal cavities that influence enzyme-substrate interactions [54]. These differences could allow them to synergize when trimming ER peptides to cover as many different sequences as possible [44]. Finally, ERAP1 and ERAP2 are polymorphic, with single-nucleotide polymorphisms that affect their functions and contribute to the variability of immune responses in natural populations [29]. Overall, ERAP1 plays a dominant role in antigen processing, whereas ERAP2 has supporting or complementary functions [55]. Figure 5 shows the structures of ERAP1, ERAP2, and IRAP.

ERAP1 polymorphism and its association with axSpA

Endoplasmic reticulum aminopeptidase 1 has variants that alter peptide-trimming activity, specificity, and expression, even if they are distant from the active site area. Different ERAP1 genetic variants have been associated with multiple HLA class I autoinflammatory diseases, such as axSpA, Behcet's disease (BD), psoriasis, multiple sclerosis (MS), type I diabetes, essential hypertension, and susceptibility to infectious diseases such as human papillomavirus (HPV)-induced cancer, HIV, hepatitis C virus (HCV), and human cytomegalovirus (HCMV) infection [42,57,58].

In 2007, the Wellcome Trust Case Control Consortium and The Australo-Anglo-American Spondylitis Consortium (TASC) Association [59] discovered that ERAP1 polymorphisms were associated with axSpA in a GWAS on Caucasian Europeans. The mechanism of action of HLA-B27 in the pathogenesis of axSpA makes it likely that peptide supply plays an important role. This process can be obtained by characterizing the peptides bound to HLA-B27 (the peptidome) [60], either through the generation or destruction of specific epitopes, affecting the role of HLA-B27 in host defense and immune homeostasis, or through alterations in the stability of the HLA-B27 molecule [61].

Reduced ERAP1 expression increases the intracellular free heavy chain (FHC) in patients with axSpA-associated HLA-B27 molecules and correlates with increased peptide length eluted from HLA-B27 molecules. This suggests that ERAP1 activity directly influences the HLA-B27 peptidome, which affects the stability of these molecules both intracellularly and at the cell surface. The levels of cell surface FHC in axSpA patients vary in response to the particular ERAP1 SNPs these patients possess, but intriguingly, they do not correlate with the trimming activity of these SNPs [62].

Alternatively, the IL-23/IL-17 axis is postulated to be involved in the pathogenesis of axSpA. The impaired function of ERAP1 and susceptibility genetic variants in ERAP1 may culminate in the accumulation of unconventional structures of HLA-B27 in the ER, which causes an unfolded protein response (UPR) in cells from axSpA patients. Unfolded protein response in macrophages from axSpA patients results in increased production of IL-23 and IL-26, which can bind to IL-23R on CD4+ T cells and induce their differentiation into IL-17-producing inflammatory Th17 cells [63]. Lee et al. [64] found an increase in IL-17 cytokines through UPR and ER stress that was not influenced by the ERAP1 gene.

**Figure 6:**
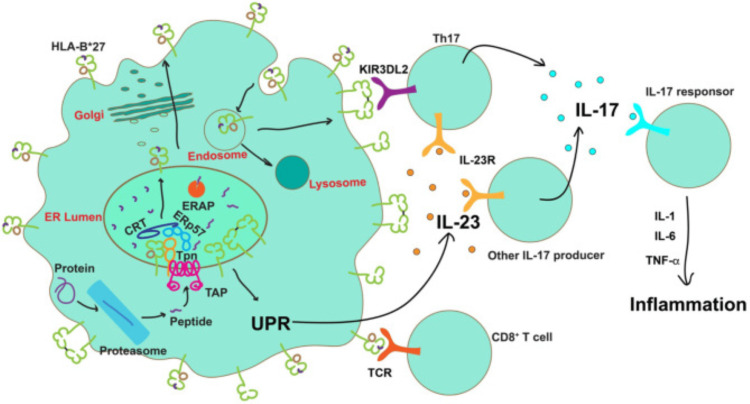
Possible role of HLA-B27 and ERAP in axSpA pathogenesis Once transported into the ER by the TAP, peptides are assembled onto nascent MHC class I molecules by the PLC, which consists of TAP, Tpn, CRT, and ERp57, before being trimmed by ERAP. The existence of specific ERAP haplotypes caused a substantial reduction in the number of peptides that were optimal for HLA-B27, which led to an accumulation of misfolded proteins in the ER or the expression of suboptimal or neoantigen-loaded HLA-B27 on the cell surface. The presence of neoantigen-loaded HLA-B27 on the cell surface triggers CD8+ T cell activation. Misfolding of proteins in the ER can trigger ER stress and initiate the UPR, which in turn leads to the secretion of IL-23. IL-23 then activates the IL-23/IL-17 axis. During HLA-B27 recycling through the endocytic pathway, free heavy chains or disulfide bond-linked homodimers of HLA-B27 are formed and expressed on the cell surface. Engagement of these aberrant species by KIR3DL2 on the surface of Th17 cells enhances their survival, proliferation, and IL-17 expression. IL-17 can promote the release of pro-inflammatory cytokines to induce inflammation [48]. PLC: Peptide loading complex, TAP: Transporter associated with antigen processing, CRT: Calreticulin, ERAP: Endoplasmic reticulum aminopeptidase, HLA: Human leukocyte antigen, ER: Endoplasmic reticulum, UPR: Unfolded protein response, axSpA: Axial spondyloarthritis, TCR: T-cell receptor, TPN: Tapasin Figure adapted from Kavadichanda et al. [48]. This article is licensed under a Creative Commons Attribution 4.0 International License, which permits unrestricted use, distribution, and reproduction in any medium, provided the original author and source are credited.

Therefore, the ERAP1 protein was highlighted in all three hypotheses regarding SpA. The arthritogenic hypothesis reveals the role of ERAP1 in cutting and regulating the sequence of antigenic peptides presented to HLA-B27. Misfolding of HLA-B27 suggests that improper peptide arrangement is the primary cause of homodimer FHC formation on the cell surface. The final hypothesis, which posits an imperfect peptide-cutting process, suggests that this can increase intracellular apoptosis and ER stress. These three hypotheses reinforce the hypothesis that cellular autoinflammatory processes play a role in axSpA development. Thus, the function of ERAP1 is crucial in axSpA disease activity because it involves the processing and regulation of antigenic peptides, which is the starting point of the autoinflammatory cascade [64,65]. Figure 6 demonstrates the possible roles of HLA-B27 and ERAP in axSpA pathogenesis.

Approximately 60% to 90% of patients with axSpA worldwide carry HLA-B27. The risk of developing AS is as high as 5% to 7% in HLA-B27-positive individuals. The genetic association of the aminopeptidase polymorphisms ERAP1 and ERAP2 with axSpA is the second strongest after HLA-B27, accounting for 15% to 25% of the population risk [37]. The genetic interaction between ERAP1 and HLA-B27 in axSpA indicates that peptide cleavage and presentation contribute to axSpA susceptibility [66]. Together, HLA-B27 and ERAP explain 70% of the genetic risk of developing SpA [37]. The bulk of ERAP1 SNPs associated with SpA are located close to the catalytic site (aa residues 346 and 349), in the binding groove (aa residues 725 and 730), or close to locations that potentially affect conformational rearrangements (aa residues 528 and 575). Further, SNPs are also found in inter-domain sites, or domain IV, a regulatory region responsible for C-terminal residue peptide binding [67]. Controversial results have been reported regarding the relationship between ERAP1 SNPs and axSpA susceptibility.

The rs27044 Polymorphism

Multiple ERAP1 features are affected in a length-dependent manner by rs27044 [68], which may lead to susceptibility to axSpA [39]. The rs27044 gene encodes the Q730E amino acid substitution, which is correlated with modifications in peptide length preference and trimming specificity [52,69,70].

In 2010, the first confirmation in a non-Caucasian population was that genetic polymorphisms in ARTS1 (SNP rs27044) were associated with axSpA, implicating common pathogenetic mechanisms in Korean and Caucasian patients with axSpA [71]. Choi et al. [72] discovered that rs27044 was associated with axSpA in Asians and Caucasians. However, a later analysis by Lee et al. found that the association only existed in the general population and Caucasians but not in Asians [73]. Wang et al. [74] found that the rs27044G allele was a predisposing factor for axSpA in Taiwanese. A meta-analysis of 26 case-control studies with 31 cohorts concluded that rs27044 is significantly correlated with axSpA in Asians and Caucasians [75]. Correspondingly, Chen et al. [76] found a significant association between rs27044 and axSpA, although their meta-analysis included only six studies with limited statistical power.

In contrast, in their meta-analysis and bioinformatics analysis, Bai et al. [77] discovered a lack of association between rs27044 and axSpA susceptibility. Similarly, the meta-analysis by Lee et al. [73] demonstrated that no association between rs27044 and axSpA could be found in Middle Easterners and East Asians, but it was found solely in Europeans. Another meta-analysis published in 2018 by Jiang et al. [78] explored the relationship between ERAP1 polymorphisms and susceptibility to axSpA in the East Asian population and found a significant difference between axSpA susceptibility and polymorphisms of rs27044.

Two Mexican studies conducted separately by Fernández-Torres et al. and Martínez-Nava et al. [79,80] reported that the rs27044 polymorphism of the ERAP1 gene was not significantly associated with axSpA. Similarly, SNP rs27044 was not reported to be associated with the disease in Spanish [81] or Chinese populations [82]. Finally, Cai et al. [83] found no association between rs27044 polymorphism and axSpA in the general population.

The rs30187 Polymorphism

The ERAP1 rs30187 SNP encodes a lysine or arginine at position 528 [84,85]. The lysine/arginine 528 substitution in biochemical assays affects ERAP1’s peptide hydrolysis activity [52]. It has been linked to autoimmune disorders in epistasis with specific MHC alleles [84,85], and modifies the set of peptides presented by MHC [68,86]. The rs30187 polymorphism, at position 1583 in exon 11, induces a substitution from C to T (R528AK), and several investigations have shown that R528 reduces the activity of ERAP1 [8,87]. The rs30187, which encodes the K528R amino acid replacement, reduces the efficacy of peptide trimming by affecting the kinetic process of modifying ERAP1 from active to inactive [69]. Its localization near the entry point of the substrate pocket could affect substrate affinity with the enzyme and decrease ERAP1 activity [88]. Owing to its lower function, the rs30187 allele, which has slower rates of peptide trimming than wild-type ERAP1 (40%), is protective [89]. Sanz-Bravo et al. [69] discovered that rs30187 influences ERAP1 activity by influencing the N-terminal flanking residues, peptide length, internal sequence, and HLA-B27 affinity.

Similar to rs27044, rs30187 is associated with axSpA in Asians and Caucasians [72]. Nonetheless, their subsequent analysis discovered that the association existed only among the general population and Caucasians but not among Asians [73]. The SNP rs30187 of ERAP1 was significantly associated with axSpA in Koreans [71] and Taiwanese [74], whereas the T/T genotype was associated with axSpA compared to the C/C genotype in the Iranian population [90]. Gao et al. [75] discovered in 2020 that, although the positive association was observed under the allelic model in Asians and Caucasians, genotypic comparisons justified the association only in Caucasians but not Asians. Similarly, Wang et al. [91] noticed that the ERAP1 SNP rs30187 had significantly different genotype and allele distributions between patients with axSpA and healthy controls.

Chen et al. supported this significant link between rs30187 and axSpA in a meta-analysis consisting of 8,530 axSpA patients and 12,449 controls [76]. Another meta-analysis by Cai et al., consisting of 24,271 axSpA patients and 42,666 controls, supported this link as well [83]. The ERAP1 rs30187 polymorphism was not associated with axSpA in the Zhejiang [92], Turkish [93], or Algerian populations [94]. Moreover, 534 Caucasian patients with axSpA and 830 healthy controls were included in a meta-analysis and bioinformatics analysis; there was no significant association between the minor allele of rs30187 and axSpA susceptibility [77].

The rs26653 Polymorphism

The ERAP1 rs26653 genotype may influence the adenosine triphosphate (ATP)-binding reserve and transport efficacy of TAP or alter the substrate specificity and proteolytic capability of the immunoproteasome [95]. Cinar et al. [93] were the first to address the relationship between ERAP1 and axSpA in a Turkish population. They confirmed that the frequency of the rs26653 SNP was higher in patients than in controls. In addition, Küçükşahin et al. [95] reported that, in a Turkish population, there was a statistically significant difference in the frequency pattern of the rs26653 SNP C/C homozygous genotype in axSpA patients, and the frequency of the rs26653 risk allele was higher in axSpA patients than in controls. In a more recent study, Wang et al. [91] examined how prevalent ERAP1 allelic variants (single nucleotide variant (SNV) haplotypes) in Taiwan affect ERAP1 function and axSpA susceptibility in the presence or absence of HLA-B27 and found that the ERAP1 SNP (rs26653G > C) had significantly different genotype and allele distributions between 863 axSpA patients and 1438 healthy controls. In contrast, an earlier study did not support the contribution of rs26653 to axSpA pathogenesis, particularly in HLA-B27-positive patients [8].

The rs27037 Polymorphism

In 2018, a meta-analysis revealed that rs27037 is significantly associated with axSpA [78]. Another meta-analysis published in 2015 found that rs27037 was positively associated with the risk of axSpA in Caucasians and Asians [83]. The same observation was obtained in a bioinformatics analysis of genetic variants of ERAP1 in axSpA; SNP rs27037 was statistically significant in a combined European and Asian study [88]. Lee and Song [73] also reported a significant association between rs27037 polymorphism and axSpA susceptibility in European and Asian populations. Furthermore, the ERAP1 rs27037 polymorphism locus is highly associated with axSpA in the Chinese population [92].

The previous finding did not agree with that of Su et al. [96], who conducted a case-control association study and meta-analysis to assess whether SNPs in ERAP1/ERAP2 and RUNX3 confer susceptibility to axSpA in Han Chinese individuals. The case-control study between HLA-B27-positive patients and healthy controls failed to demonstrate an association between rs27037 and axSpA. Moreover, the meta-analysis revealed no association between rs27037 in ERAP1 and the disease. Tang et al. [97] also examined the association between five polymorphisms in the ERAP1 gene and the risk of axSpA in a Chinese population; they failed to provide evidence for an association between rs27037 polymorphisms in ERAP1 and axSpA risk. Zhang et al. [84] could not confirm the association of axSpA with ERAP1 SNP rs27037. Likewise, a Turkish study by Akbulut et al. [98] found no risk association between axSpA and rs27037 polymorphism in the studied population.

The rs27434 Polymorphism

Li et al. [99] conducted a case-control association study to determine whether ERAP1 is also associated with the incidence of axSpA in a Chinese population and whether it is correlated with clinical features. Their results showed that SNP rs27434 was significantly associated with the disease, which is consistent with the results of Liu et al. [92], who found that the locus of the ERAP1 rs27434 polymorphism was at a high significance level with axSpA in the Zhejiang population. Another study confirmed a weak association between ERAP1 rs27434 and axSpA in the Beijing Han Chinese population [84]. In Iran, the rs27434 G/G genotype was found to be inversely associated with axSpA compared to the A/A genotype [90].

The rs27980 Polymorphism

In a recent study, Wang et al. [91] discovered that the ERAP1 intron SNP (rs27980A > C) had significantly different genotype and allele distributions between patients with axSpA and healthy controls. Similarly, Liu et al. [92] found that the locus of the ERAP1 rs27980 polymorphism was at a high significance level with axSpA in the Zhejiang population. The rs27980C allele appears to be a modest risk factor for axSpA susceptibility in Taiwanese individuals [74]. Nevertheless, Zhang et al. and Cinar et al. [84,93] could not confirm the association with axSpA using SNP rs27980 in Turkish and Beijing Han Chinese populations, respectively.

The rs10050860 Polymorphism

Recently, genotype T of the rs10050860 SNP of the ERAP1 gene was found to have a protective effect on axSpA in the population of Western Algeria [94]. The same conclusion has been reported in Korean [71], Turkish [93], and Zhejiang populations [92]. Likewise, this finding was consistent with Bai et al.'s [77] meta-analysis and bioinformatics analysis.

The rs17482078 Polymorphism

The ERAP1 SNP rs17482078 showed findings similar to those of rs10050860. It has a protective effect with respect to axSpA in the Korean [71], Turkish [93], and Zhejiang populations [92]. According to Bai et al. [77], there is no significant association between the minor allele of rs17482078 and axSpA susceptibility.

The rs2287987 Polymorphism

The rs2287987 (Met349Val) is located close to the catalytic center and affects enzyme activity [100]. A GWAS performed on British individuals first described the association of ERAP1 rs2287987 with susceptibility to axSpA [59]. The association between these polymorphisms and AS was observed in Spanish [81], Portuguese [101], Hungarian [102], Polish [103], and Iranian [42] populations. Another meta-analysis showed that rs2287987 seems to be associated with AS in Caucasians and overall populations but not in Asians [83]. Bai et al. [77] discovered that the minor allele of rs2287987 is a protective factor against axSpA in the HLA-B27-positive population. Furthermore, there was no significant association between rs2287987 and axSpA in the Korean [71] and Turkish populations [93].

The rs27037 Polymorphism

Evidence for the involvement of SNP rs27037 in axSpA has been demonstrated in several studies [74,91,92]. This involvement could not be confirmed in the Turkish [93,98] and Beijing Han Chinese populations [84].

ERAP1 and HLA-B27 interactions

Endoplasmic reticulum aminopeptidase 1 may function in tandem with HLA-B27 molecules, and ERAP1 polymorphisms may result in an abnormal peptide-HLA (pHLA)-B27 repertoire linked to pathogenic immune responses. Furthermore, abnormal pHLA can be unstable and misfolded [104]. Misfolded HLA-B27 molecules can cause ER stress or appear as surface HLA class I-free heavy chains (FHCs), resulting in abnormal immune interactions with various receptors [105-108]. Direct or indirect changes in the ERAP1-HLA-B27 interaction could be critical, causing changes in peptide presentation, creating free heavy chains by HLA-B27 molecules, and contributing to differential subtype associations in the SpA [109].

In their review, Zambrano-Zaragoza et al. [110] demonstrated a consistent association between ERAP1 and axSpA in HLA-B27-positive cases. Abnormal peptide trimming or presentation of ERAP1 and HLA-B27 play a role in the pathogenesis of HLA-B27-associated axSpA. Wang et al. [91] found that the allele distributions of ERAP1 SNPs (rs26653, rs26618, rs30187, rs469783, rs27044, and rs27037) differed significantly between HLA-B27-positive patients and HLA-B27-negative patients. Wang et al. [74] examined whether ERAP1 SNPs are linked to axSpA susceptibility and disease severity in Taiwanese individuals. They demonstrated that ERAP1 SNPs are associated with HLA-B27 positivity in Taiwanese patients with axSpA. These findings support the idea that ERAP1 and HLA-B27 play complementary roles in axSpA pathogenesis in humans. Their results also indicated that abnormal antigen processing by ERAP1 and antigen presentation by HLA-B27 may be essential pathways in the development of axSpA. In contrast, patients with axSpA who are negative for HLA-B27 may develop pathological immune responses via other unidentified biological pathways.

Several studies [84,89,92,95,110-113] confirmed an epistatic interaction between the ERAP1 SNPs and the HLA-B27 loci. This interaction was not discovered by Bai et al., who found no significant association between the minor alleles rs30187 and rs10050860 and axSpA susceptibility in an HLA-B27-positive population in a recent meta-analysis [77]. Similarly, Asmaa et al. [94] investigated the roles of rs30187 and rs10050860 in the presence and absence of HLA-B27. They found no link between HLA-B27 positivity or negativity and the frequency of ERAP1 SNPs (rs30187 and 10050860). Similarly, Cinar et al. [93] found no association between HLA-B27 positivity and the ERAP1 genotype frequency distribution. Again, no significant association was found between the frequency of ERAP1 and that of B27:05 or B27:02:01. Similarly, Zhang et al. [84] found no correlation between ERAP1 SNP rs27037 and axSpA in either HLA-B27-negative or HLA-B27-positive axSpA groups.

Association of ERAP1 SNPs with axSpA clinical parameters

Recognizing the genetic variables that affect functional severity would improve functional status prediction in patients with axSpA. The immune system and bone development share cellular and molecular signaling pathways that regulate hematopoietic cells and bone homeostasis [114]. According to animal models, inflammation and new bone formation are not related [115,116]. Clinically, various anti-TNF drug treatments repress inflammation but do not slow structural development, according to the modified Stoke ankylosing spondylitis spinal score (mSASSS) [117,118]. These findings suggest that syndesmophyte development is likely attributable to the intrinsic genetic effects of ERAP 1 on the p/MHC class I complex formation [74].

Szczypiorska et al. [81] were the first to report a link between SNPs in ERAP1 and the axSpA functional status. Likewise, Wang et al. [74] discovered that the SNPs rs27044 and rs30187 were linked to syndesmophyte formation. Both studies have suggested that ERAP1 is related to disease severity.

In an Iranian cohort, rs10050860 was strongly associated with the bath ankylosing spondylitis functional index (BASFI) score [42]. The SNP rs27044G and SNP rs30187T allele carriers are prone to developing syndesmophytes in axSpA patients, indicating that ERAP1 cSNPs may affect axSpA disease severity. After controlling for HLA-B27 positivity, SNP rs30187 remained significantly associated with syndesmophyte formation, whereas SNP rs27044 was marginally associated. The SNPs rs27037 and rs27980 were not significantly associated [74].

Küçükşahin et al. [95] found that the mean bath ankylosing spondylitis disease activity index (BASDAI), BASFI, bath ankylosing spondylitis metrology index (BASMI), and ankylosing spondylitis disease activity score-C-reactive protein (ASDAS-CRP) values were greater among those with the ERAP1 rs26653 C/C SNP genotype than other patients; the differences were statistically significant. However, they reported similarities between patients with different rs26653 SNP genotypes (C/C, C/S, or G/G) regarding the frequency distributions of many clinical (presence of peripheral arthritis or uveitis) and demographic (sex, family history of SpA, age at disease onset) characteristics, except for individuals with enthesitis, who had the rs26653 C/C SNP genotype more frequently than those without it. Li et al. [99], in their case-control association study, concluded that except for the SNP rs27510, which was significantly correlated with onset age in patients with axSpA, none of the examined ERAP1 SNPs showed significant association with the demographic and clinical measurements of axSpA (age, sex, family history, onset site, dactylitis, peripheral arthritis, hip joint involvement, iritis, enthesitis, ESR, and CRP). Bugaj et al. [119] found that patients with the ERAP1 rs2287987 AA genotype more frequently presented with enthesitis; in addition, ERAP1 rs2287987 affected the initial CRP value among Polish patients, but this relationship was not statistically significant after Bonferroni correction.

In contrast, Asmaa et al. [94] found no differences in BASDAI, BASFI, or CRP levels in patients with axSpA carrying rs30187 and rs10050860 polymorphisms. Nossent et al. [120] investigated the role of ERAP1 variants in the axSpA clinical phenotype. They discovered that the ERAP1 rs27044/rs30187 haplotype C/T is associated with a lower risk of extraspinal disease and systemic inflammation in Nordic patients with axSpA. However, no link was found between the ERAP1 haplotype and proinflammatory cytokine levels.

The SNPs of the ERAP1 gene correlated with the mSASSS and showed no correlation with the ASDAS-erythrocyte sedimentation rate (ASDAS-ESR). Significant differences were observed in the SNP ERAP1 gene on ERAP1 and IL-17A levels in subjects with lipopolysaccharide and IFN-γ induction, but no significant difference was observed in IL-23 levels [121]. In a Portuguese axSpA cohort, Pimentel-Santos et al. [122] found no association between ERAP1 SNPs (including rs27044 and rs30187) and BASDAI, BASMI, mSASSS, or disease duration. Furthermore, Cinar et al. [93] discovered that the rs26653 SNP linked to axSpA risk was unrelated to disease activity or functional scores in a Turkish population. Additionally, no significant associations were found between carriages of the allele rs27044 and sex, past or present peripheral arthritis, age at first complaint, years between these first complaints, or the diagnosis of axSpA [123].

A French study [124] investigated the association between SNPs located in ERAP1 and the sacroiliac joint (SIJ) and spinal MRI inflammation in early-onset SpA. One SNP located in ERAP1 (rs27434) and the haplotype CCT of ERAP1 were associated with SIJ inflammation detected by MRI, but these associations were below the Bonferroni-corrected threshold of significance. In contrast, no relationship was found between ERAP1 SNPs (rs30187, rs27044, rs27434, rs17482078, rs10050860, and rs2287987) and axSpA activity as measured by SIJ inflammation on MRI, BASDAI score, ASDAS-CRP, and CRP in the French population.

## Conclusions

The role of HLA-B27 in axSpA pathogenesis is unclear. However, over the past decade, ERAP1 has been shown to play an important role. Identifying additional non-MHC susceptibility loci for axSpA, such as ERAP1, is of particular interest because it highlights the critical biological pathways involved in SpA pathogenesis, which may have a potential therapeutic impact. Modulating ERAP1 function through the design of inhibitors may be a vital tool for changing immune responses in SpA. The effect of ERAP1 polymorphisms on susceptibility to axSpA may vary among ethnic groups. The epistatic interaction between ERAP1 SNPs and the HLA-B27 loci in axSpA pathogenesis has been confirmed in several studies. However, this interaction requires further investigation in different ethnicities. The association of ERAP1 SNPs with the clinical assessment of axSpA is inconsistent for various disease-activity parameters. Therefore, it is crucial to identify ERAP1 genetic variations in different populations to gain insight into its role in the susceptibility and severity of axSpA.
